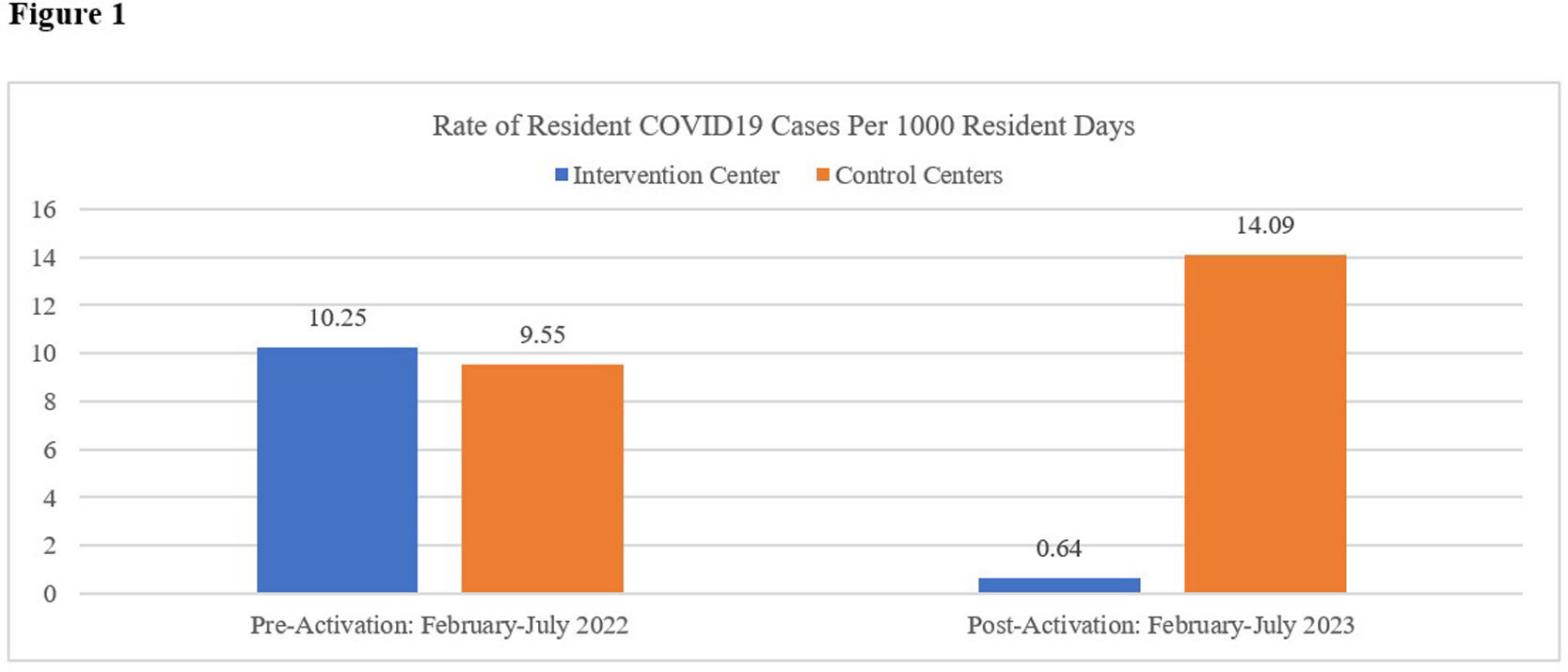# Sustained Microbial Burden Reduction and Impact on Covid19 Cases in Long-Term Care Facility through Advanced Photocatalysis

**DOI:** 10.1017/ash.2024.260

**Published:** 2024-09-16

**Authors:** Kim Trosch, Amy Carenza, Deborah Birx, Julie Britton, Charmarie Adkins

**Affiliations:** ActivePure Technologies; Genesis HealthCare

## Abstract

**Background:** COVID19 remains deadly to Americans over 75 years old despite vaccination and additional infection control practices in long term care (LTC). The evolution of more transmissible COVID19 variants and continued viral aerosols result in persistent COVID19 outbreaks in LTC during high community levels of COVID19. Despite the end of pandemic Federal support and the continued vulnerability of elderly to the virus, LTC facilities remain dedicated to protecting this vulnerable population. The study hypothesized that utilization of continuous, facility-wide, advanced photocatalysis (AP) disinfection technology will reduce microbial burden in air and on surfaces, demonstrating a decrease in infectious aerosols and subsequent COVID19 cases among residents and workers. **Methods:** A prospective facility controlled experimental study was performed in skilled nursing facilities in Pennsylvania and New Jersey from January 2023 to April 2023 to surveil aerobic bacterial and fungal colony forming units (CFUs) in air, and Methicillin-resistant Staphylococcus aureus (MRSA) and fungal CFUs on surfaces and floors prior to and post AP technology installation. Impacts on resident COVID19 cases were recorded and compared to the same extended observation period (February-July 2023) one year prior (2022) with similar year over year community COVID19 rates. In addition, two matched control centers in regional proximity to the intervention facility were also prospectively studied. A one-way analysis of variance (ANOVA) was used to analyze mean microbial burdens after each post activation period (significance p <.05). **Results:** From baseline to final testing, the intervention facility surface testing showed a 93% reduction in mean aerobic bacterial CFUs (p=.002); 96% reduction in mean fungal CFUs (p<.001); 97% reduction in mean MRSA CFUs (p<.001). Floor testing also showed reductions in mean CFUs for aerobic bacteria by 92% (p<.001); 96% for fungi (p<.001); 99% for MRSA (p<.001). Air testing showed reductions in mean CFUs for aerobic bacteria by 87% (p=.005); 36% for fungal (p=.005). The intervention facility observed a 94% reduction in resident COVID19 cases compared to the matched control facilities that increased 46% during the 2023 time period (Figure 1). **Conclusion:** This study is on the pioneering edge of demonstrating that continuous and persistent disinfection technology reduces contaminant reservoirs on surfaces, floors, and air and clearly decreases infectious aerosols and improves resident outcomes by dramatically reducing COVID19 transmission in LTC facilities.

**Figure:**